# Editorial: Brain-inspired computing: from neuroscience to neuromorphic electronics for new forms of artificial intelligence

**DOI:** 10.3389/fnins.2025.1565811

**Published:** 2025-02-11

**Authors:** Daniela Gandolfi, Jonathan Mapelli, Francesco Maria Puglisi

**Affiliations:** ^1^Department of Engineering “Enzo Ferrari,” University of Modena and Reggio Emilia, Modena, Italy; ^2^Center for Neuroscience and Neurotechnology, University of Modena and Reggio Emilia, Modena, Italy; ^3^Department of Biomedical, Metabolic and Neural Sciences, University of Modena and Reggio Emilia, Modena, Italy

**Keywords:** artificail intelligence (AI), neuromorphic, neuroscience, electronics, brain-inspired

The increasing diffusion of AI applications into daily life led to a significant rise in demand for advanced machine learning systems, such as artificial neural networks, which now outperform humans in many tasks. The rapid growth of generative AI solutions based on transformer architecture (Vaswani et al., 2017) has further accelerated the need for more powerful computational hardware. Additionally, research in humanoid robotics has focused on developing systems that replicate neural processes. However, conventional hardware solutions are unsustainable, as they require frequent training cycles, supervised learning, and large offline datasets which constrain the adoption of sustainable AI. Recently, industrial applications using conventional models have emerged, but neuromorphic approaches, inspired by the brain's functioning, offer promising, sustainable alternatives (Bhanja et al., 2023). Neuromorphic is an umbrella term that spans many interdisciplinary fields, including neuroscience, material science and electronic architectures, extending into mathematical and software models. Advances in computational neuroscience, along with the development of neuronal and synaptic models have driven the emergence of neuro-inspired microelectronics. Firstly, the proposed circuits were primarily based on the observation that transistors operating in the sub-threshold regime share remarkable similarities with the biophysics of biological neuronal membranes (Indiveri et al., 2011). This paved the way for the development of novel architectures based on silicon neurons. The maturity of the CMOS process allowed the steady implementation of brain-machine interfaces and neuro-inspired low-power computation systems, achieving higher levels of complexity (Indiveri et al., 2011). However, more recently the scientific community acknowledged the superior performance of new materials and emerging devices in mimicking neuronal behaviors, further accelerating the research in this direction. Notable examples are functionalized nanomaterials (Sangwan and Hersam, 2020; Kim et al., 2020) and memristive devices (Zanotti et al., 2021; Lanza et al., 2022), which have demonstrated the ability to replicate synaptic plasticity through long- and/or short-term changes in synaptic efficacy (Florini et al., 2024). As these new solutions stabilize and move toward commercial viability, architectures based on them are emerging mainly in the form of Spiking Neural Networks (SNN) that outperform traditional platforms in distributed computation, showing higher energy efficiency (Lanza et al., 2022). Interestingly, an emerging domain of theoretical and computational neuroscience, based on a Bayesian approach adopted to model brain functions (Gandolfi et al., 2022), recently opened promising perspectives in terms of energy efficient neuromorphic applications.

Much of the focus in the development of neuromorphic solutions has been on the hardware. Conversely, on the software side, efforts were mainly aimed at creating AI algorithms inspired by neuronal architectures. Despite the recent increase in publications on AI solutions based on artificial neural network (ANN) and the recognized success of generative AI machineries, there is a growing consensus that alternative approaches must be investigated and implemented. This is due to the unsustainability of the current approach, that is evidently too resource-hungry (i.e., it is associated with unbearable energy and water consumption, as well as land use) (Obringer et al., 2021). In this respect, the brain, due to its event-based communication, remains the key model to emulate by virtue of its remarkable computational power despite its limited energy resources.

In this fast-paced growth context, significant research efforts are often carried out within individual scientific domains. However, future breakthroughs are likely to come from cross-domain research encompassing many sectors such as neuroscience, electronics, computer science, and robotics, all driven by the same underlying goals and foundational principles. This joint Frontiers in Neuroscience and Frontiers in Electronics topic aims at showcasing the latest advancements in neuromorphic computing and fostering reciprocal contaminations.

Such an attempt is shown in the work by Bouanane et al., in which the authors explore the impact of synaptic and membrane leakages in spiking neurons. By comparing three neural models with different computational complexities using feedforward and recurrent topologies for event-based visual and auditory pattern recognition, the authors demonstrated that leakages significantly affect accuracy, particularly when temporal information is present in the data and explicit recurrence is incorporated in the network. Advances in SNNs are shown also in the work by Wang et al., in which the authors propose a brain topology-improved SNN for efficient reinforcement learning. Starting from topologies generated and selected as subsets of the Allen mouse brain connectome, three key topology candidates are identified and integrated with the hybrid numerical solver-improved leaky-integrated-and-fire neurons. In a series of four animal-survival-inspired reinforcement learning task, the authors show that their BT-SNN can achieve higher scores than the conventional SNN and some classical ANNs. Research on SNNs is then shifted toward the architecture level in the work by Bittar and Garner, in which the authors present a physiologically inspired speech recognition architecture, and demonstrate that end-to-end gradient descent training leads to the emergence of neural oscillations in the central SNN. Furthermore, they emphasize the critical role of feedback mechanisms, such as spike frequency adaptation and recurrent connections, in regulating and synchronizing neural activity to improve recognition performance. Along the same line, Okonkwo et al. show the results of their research on SNN oriented toward mobile, low-cost, and energy-aware smart circuits. Their work introduces a novel bio-inspired reinforcement learning system architecture that offers significant energy savings without compromising real-time autonomous processing or the accuracy required for context-dependent tasks. The hardware architecture, synthesized, simulated, and tested on Intel MAX10 FPGA, successfully models features analogous to synaptic tagging, changes in the exploration schemes, synapse saturation, and spatially localized task-based activation observed in the brain. Yin et al. further advance the research on SNNs toward applications, as they investigate neuromorphic image sensors that draw inspiration from the biological retina to implement visual computations in hardware. Specifically, they present a technology-circuit co-design solution that implements object motion sensitivity and looming detection at the retina's output. Simulations using Global foundries 22 nm technology show that the proposed retina-inspired circuits can be fabricated on image sensing platforms in existing semiconductor foundries. A key aspect of neuromorphic computing is addressed in the review by Borghi et al. in which the authors point out that, although efforts to address neuromorphic solutions through hardware based on top-down CMOS-based technologies have obtained interesting results in terms of energetic efficiency improvement, the replication of brain's self-assembled and redundant architectures is not considered in the roadmaps of data processing electronics. In their review, the authors discuss possible directions in terms of hybrid hardware solutions where self-assembled substrates coexist and integrate with conventional electronics. Finally, the mini-review of Liu et al. examines three application scenarios of brain-machine interfaces in the metaverse: generative art, serious gaming for healthcare, and brain-machine interface applications for facial expression synthesis in the virtual society. It investigates existing commercial products and patents, draws analogies with the development processes of network security and neurosecurity, bioethics and neuroethics, and discusses the challenges and potential issues that may arise when brain-machine interfaces mature and become more widely applied.

We are grateful to the authors for their outstanding contributions to this Research Topic and hope that this Research Topic marks the beginning of a series of cross-disciplinary studies on brain-inspired computing, integrating both software and hardware, while also bridging conventional AI with bio-inspired systems. We look forward to further advancing the discussion in this field.
